# Histopathological Features of Dental Pulp in Teeth with Different Levels of Chronic Periodontitis Severity

**DOI:** 10.5402/2012/271350

**Published:** 2012-04-10

**Authors:** Elizangela Partata Zuza, Ana Luiza Vanzato Carrareto, Raphael Carlos Comelli Lia, Juliana Rico Pires, Benedicto Egbert Corrêa de Toledo

**Affiliations:** Department of Master of Dental Science, School of Dentistry, Educational Foundation of Barretos (UNIFEB), Avenida Roberto Frade Monte, 389, 14783-226 Barretos, SP, Brazil

## Abstract

*Purpose*. To evaluate the histopathological condition of the pulp in teeth with different levels of chronic periodontitis in humans. *Methods*. Twenty-five single-root nondecayed teeth were divided into three groups as follows: group 1, clinical attachment level (CAL) 3 to 4 mm and alveolar bone loss (BL) from 4 to 6 mm without reaching the tooth apex; group 2, CAL ≥ 5 mm and BL > 6 mm without reaching the tooth apex; group 3, CAL ≥ 5 mm and BL > 6 mm up to the tooth apex. Histological analyses were accomplished after laboratorial processing. *Results*. The mean of CAL was 3.2 ± 0.7 mm in group 1, 7.6 ± 2.0 mm in group 2, and 12.1 ± 2.8 mm in group 3, while for BL it was 4.8 ± 0.9 mm, 7.6 ± 2.2 mm, and 11.9 ± 2.1 mm, respectively. Histopathological data in the pulpal chambers were similar among the three groups showing normal aspects, and, the radicular pulps showed variable levels of reactive dentin, fibrosis, dystrophic mineralizations, atrophy, and mononuclear inflammatory infiltrate. *Conclusions*. Gradual progression of the chronic periodontitis led to changes in the histopathological aspects of the radicular pulp with progressive involvement.

## 1. Introduction

There are communications between the pulpal tissues and the periodontal structures [1, 2]. Some reports showed the effects of pulpal alterations on the periodontium [3–5]; however, few studies evaluated the influence of periodontitis upon pulpal tissues [3]. The microbiota of both lesions seems to be similar, but the developments of models are needed for better histological and clinical investigation [6–8].

The influence of the periodontitis upon pulpal tissues happens not only when there is a tooth apex involvement [9, 10], but also in cases that the periodontitis does not reach the tooth apex [11, 12]. Radicular dentin of teeth with periodontitis can be invaded by putative periodontal pathogens, such as *Prevotella intermedia*, *Phorphyromonas gingivalis*, *Fusobacterium nucleatum*, *Bacteroides forsythus*, *Peptostreptococcus micros*, and *Streotococcus intermedius* [13].

Previous studies [14, 15] have also demonstrated that the bacterial invasion can occur in the root cementum and radicular dentin of those teeth with periodontal involvement, and bacteria were detected on the pulpal wall and in dental pulp of those teeth, which shows the influence of the periodontitis upon pulpal tissues. There is a scarceness of histological studies that have assessed the influence of different degrees of periodontitis upon the pulp tissue. Thus, the aim of this study was to evaluate the pulpal histopathological conditions in teeth with different levels of chronic periodontitis in humans.

## 2. Materials and Methods

The present study was approved by the Ethics Committee of the Educational Foundation of Barretos (protocol no. 02/13/11/2007). Patients were included in the study after signing an informed consent form. Thirty-seven single-root teeth indicated for extraction were evaluated in 16 patients (8 of each gender; mean age of 43.2 years), who sought treatment at dental clinics of UNIFEB. The teeth showed clinical features of chronic periodontitis, but with no signs of caries, abrasions, erosions, attritions, or restorations. They also should not have been submitted to periodontal therapy in the last 6 months. Of the 37 teeth extracted, 25 of them were included in the study and 12 were discarded for presenting initial caries lesions, small composite restorations, erosions, or abrasions.

### 2.1. Periodontal Evaluation

The periodontal parameters were evaluated, as follows: probing depth (PD); gingival recession (GR); clinical attachment level (CAL) [16]; extension of alveolar crest bone loss (BL). The PD was defined as the distance from the gingival margin to the base of the sulcus or periodontal pocket. GR was pointed out as the distance from cementoenamel junction and the most apical point of the gingival margin. CAL was considered as the distance from the cementoenamel junction to the base of the sulcus or periodontal pocket. BL was defined as the distance from the cementoenamel junction to the most coronal point of bone crest. The periodontal measurements of PD, GR, and CAL were recorded at six sites on each tooth (distobuccal, buccal, mesiobuccal, distolingual, lingual, and mesiolingual) by means of a type-Williams periodontal probe (Hu-Friedy PCPN115BR). The assessment of BL was carried out in radiographs taken by the parallelism periapical technique by means of a precision pachymeter, with an appropriate light box in a dark environment.

 The teeth were divided into three groups considering CAL parameters [17] and BL criteria, as follows: group 1, moderate periodontitis with clinical attachment level (CAL) 3 to 4 mm and alveolar crest bone loss (BL) from 4 to 6 mm without reaching the tooth apex; group 2, severe periodontitis with CAL ≥ 5 mm and BL > 6 mm without reaching the tooth apex; group 3, serious periodontitis with CAL ≥ 5 mm and BL > 6 mm up to the tooth apex (Table 1). Each tooth showed at least 4 points with periodontal involvement to be included in each group.

### 2.2. Histological Evaluation

After exodontics, the teeth were superficially cleaned with saline solution (0.9% NaCl) and the pulpal chamber was perforated for exposure. The teeth were kept in plastic containers and immersed into Buffered Formol (pH 7.4) for fixation. Later, they were decalcified and submitted to the laboratorial protocol for semisequenced sections of 5 *μ*m in buccal/lingual direction, as well as dyeing with hematoxylin and eosin. Histological assessment of the specimens was performed to evaluate structural aspects of dental pulp, such as presence of reactive dentin, fibrosis, dystrophic mineralizations, atrophy, and mononuclear inflammatory infiltrate.

## 3. Results

### 3.1. Periodontal Clinical Data

The group with serious periodontitis (group 3) showed the highest measures of CAL and BL compared to the other groups (*P* < 0.05), whereas groups 1 (moderate periodontitis) and 2 (severe periodontitis) did not show significant differences between each other (*P* > 0.05). There were no significant differences in the GR and PD parameters between groups 1 and 2 and also between groups 2 and 3 (*P* > 0.05); nevertheless, a significant difference was verified between groups 1 and 3 (*P* < 0.05). Periodontal clinical data are shown in Table 2.

### 3.2. Histological Aspects

**Group 1**

**Figure 1:**
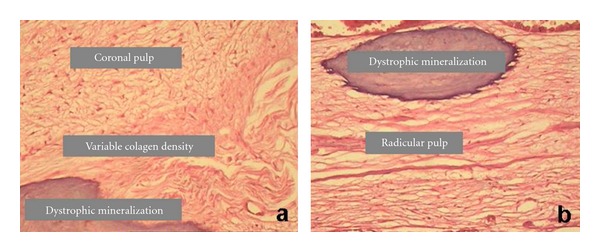
Histological aspects of (a) coronal pulp and (b) radicular pulp in group 1.

The coronal portion of the pulp of all teeth showed loose connective tissue of normal appearance, with predominance of fibroblasts, collagen, dentin, predentin, and odontoblastic layer (Figure 1(a)). The root portion revealed reduction of the pulp canal space due to an appositional increment of reactive dentin following a repair pattern, with a decrease in the number and size of the odontoblasts. Reactive dentin showed an irregular pattern, characterized by the arrangement of dentinal tubules with odontoblastic prolongment and variations in the globular mineralization. The connective tissue of the radicular pulp revealed an increment in the collagen evolution, and some needle-shaped elongated and discretely diffuse mineralization were verified in some specimens (Figure 1(b)).

**Group 2**

**Figure 2:**
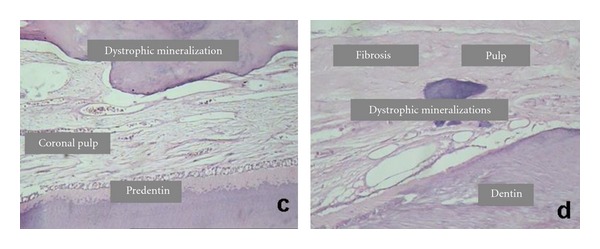
Histological aspects of (c) coronal pulp and (d) radicular pulp in group 2.

The coronal portion of the pulp showed similar aspects to group 1 (Figure 2(c)). Reactive dentin in a more repairing and irregular pattern was verified in the root pulp, with little canalicular arrangement and decrease in the number and size of underlying odontoblasts. There was a predominance of collagen fibers bundles arranged in the connective tissue, with fibroblasts and fibrocytes. Dystrophic mineralization was observed along the root canal. Inflammatory infiltrate was observed with mononuclear cell predominance in the connective tissue of the root pulp (Figure 2(d)).

**Group 3**

**Figure 3:**
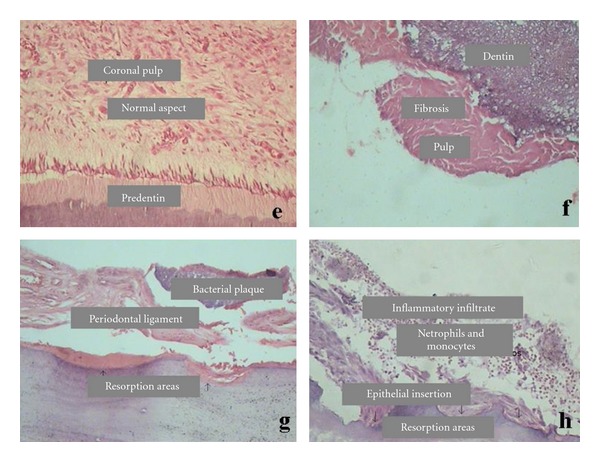
Histological aspects in group 3. (e) Coronal pulp, (f) radicular pulp with fibrosis, (g) resorption areas and breakdown of the periodontal ligament and (h) inflammatory infiltrate and resorption areas.

The aspects of coronal pulp were not different from those observed in groups 1 and 2 (Figure 3(e)). Pulpal fibrosis represented by a great amount of collagen fibers and less cellularity was verified in the root pulp (Figure 3(f)). Reactive dentin pattern revealed varied levels, showing irregularities in the arrangement of canaliculi and mineralization, as well as pulp atrophy. Dystrophic mineralization was verified along the root canal, showing as elongated, scattered, nodular, and irregular amorphous structures. Some specimens showed internal resorption. Inflammatory infiltrate showed predominance of mononuclear cells (lymphocytes, plasmocytes, and macrophages) and a varying number of polymorphonuclear leukocytes. Some specimens revealed angioblastic proliferation with agglomeration of plasmocytes (Figures 3(g) and 3(h)).

## 4. Discussion

Our histological results revealed that the coronal pulp showed normal aspects in the three groups, while radicular pulp presented a gradual increasing in histological changes. Histological studies [18–22] confirmed that pulpal alterations can occur in the cases of periodontitis, with dystrophic calcification, atrophy, cell number reduction, fibrosis, repair dentin formation, inflammation, degeneration, and pulpal tissue necrosis. Some findings have also shown a significant increase in the amount of dentin formed in teeth with periodontal involvement compared to healthy teeth [19, 23, 24].

 A histological study [10] showed that pulpal alterations occurred more frequently in the apical region in teeth with periodontal involvement reaching the tooth apex, while the coronal pulps showed normal features. It could be suggested that the period of progression of the periodontitis may be an important factor to promote pulpal alterations, since chronic periodontitis features a slower and long-lasting development. Furthermore, it was verified fibrosis, secondary dentin, atrophy, dystrophic calcification, and inflammatory infiltrate, which occurred more frequently in the apical region of root pulp.

Our findings showed that the pulpal changes occurred only in the radicular pulp and not in the coronal pulp, in agreement with some studies [10, 18–21]. Other authors [22] have also verified pathological alteration in the pulpal tissue, but a total degeneration was verified only when the circulatory pathway of the main canal was affected. Additionally, single-root teeth with periapical lesions were significantly correlated with deeper periodontal pockets and greater radiographic bone loss [25], which also confirms our results that showed greater clinical attachment loss and greater alveolar bone loss in the group with serious periodontitis reaching the tooth apex.

Conversely, other studies [26] did not find pulpal alterations even in cases of periodontitis reaching the tooth apex. This is in line with other studies [27, 28] that did not find correlation between the severity of periodontitis and the morphological alterations in the pulp tissue. Czarnecki and Schilder [27] verified that pulps of the teeth with periodontitis showed normal histological aspects, regardless of periodontal disease severity. Mazur and Massler [29] reported that the periodontal disease does not affect the pulp, suggesting that the pulpal degeneration was related to systemic factors. Bergenholtz and Lindhe [30] verified in monkeys that although 30% to 40% of the teeth presented clinical attachment loss, 70% of the roots showed no pulpal pathological alterations.

The influence of the pulp on the periodontal structures is more evident than the influence of periodontitis on the pulp tissue [31]; however, a chronic-degenerative reaction and asymptomatic pulpal necrosis may occur when a frequent stimulus with low intensity and long lasting was applied [32]. There is a scarceness of studies describing the histological features in healthy dental pulp in the different ages [10]. Histological observations of the influence of periodontitis on dental pulp must be added to current clinical studies [33–35]. Thus, further studies are suggested to clarify the complex relationship between the pulp and the periodontium.

## 5. Conclusions

Within the limits of this study, it was possible to conclude that a gradual progression of the chronic periodontitis led to changes in the histopathological features of the radicular pulp with progressive involvement, but not in the pulp chamber.

## Figures and Tables

**Table 1:** Groups and criteria for the study.

Groups	Number of teeth	Criteria
Group 1: moderate periodontitis	5	3 to 4 mm of attachment and radiographic bone level loss (4 to 6 mm) without reaching the apex

Group 2: severe periodontitis	10	≥5 mm of attachment and radiographic bone level loss (>6 mm) without reaching the apex

Group 3: serious periodontitis	10	≥5 mm attachment and radiographic bone level loss (>6 mm) reaching the apex

**Table 2:** Clinical results in the different experimental groups.

Clinical parameters	Group 1	Group 2	Group 3	Values of *P*
GR (mm)	2.9 ± 1.2	4.6 ± 1.0	6.0 ± 2.2	*P*_1 × 2_ = NS
				*P*_1 × 3_ ≤ 0.05
				*P*_2 × 3_ = NS

PD (mm)	1.6 ± 0.6	3.4 ± 1.7	6.0 ± 2.9	*P*_1 × 2_ = NS
				*P*_1 × 3_ ≤ 0.05
				*P*_2 × 3_ = NS

CAL (mm)	3.2 ± 0.7	7.6 ± 2.0	12.1 ± 2.8	*P*_1 × 2_ = NS
				*P*_1 × 3_ ≤ 0.05
				*P*_2 × 3_ ≤ 0.05

BL (mm)	6.3 ± 2.0	7.6 ± 2.2	11.9 ± 2.1	*P*_1 × 2_ = NS
				*P*_1 × 3_ ≤ 0.05
				*P*_2 × 3_ ≤ 0.05

mm: millimeters; GR: gingival recession; PD: probing depth; CAL: clinical attachment level; BL: bone lost; NS: nonsignificant (*P* > 0.05, Kruskal-Wallis Test).
